# Measles Induced Encephalitis: Recent Interventions to Overcome the Obstacles Encountered in the Management Amidst the COVID-19 Pandemic

**DOI:** 10.3390/diseases10040104

**Published:** 2022-11-17

**Authors:** Mufaddal Najmuddin Diwan, Saba Samad, Rabeea Mushtaq, Alifiya Aamir, Zoha Allahuddin, Irfan Ullah, Rifayat Ullah Afridi, Aneela Ambreen, Adel Khan, Nimra Ehsan, Zoia Ehsan Khattak, Antonio Ventriglio, Domenico De Berardis

**Affiliations:** 1 Dow Medical College, Karachi 75700, Pakistan; 2 Department of Pediatrics, Naseer Teaching Hospital, Peshawar 25120, Pakistan; 3 Kabir Medical College, Gandhara University, Peshawar 25120, Pakistan; 4 Khyber Medical College, Peshawar 25120, Pakistan; 5 Department of Psychiatry, University of Foggia, 71121 Foggia, Italy; 6 Department of Psychiatry, Azienda Sanitaria Locale 4, 64100 Teramo, Italy; 7 School of Nursing, University of L’Aquila, 67100 L’Aquila, Italy; 8 International Centre for Education and Research in Neuropsychiatry, Samara State Medical University, 443100 Samara, Russia

**Keywords:** encephalitis, measles, COVID-19, prevention, treatment

## Abstract

Encephalitis, a well-known complication of measles, is inflammation of the brain parenchyma which is mostly due to the viral invasion of neurons. It presents with a variety of symptoms ranging from mild to severe depending on the extent of the damaged neurons. The diagnosis is based on clinical symptoms such as fever, headache, altered level of consciousness, focal neurological deficits, etc. A detailed history and physical examination facilitate the diagnosis. Investigations include blood tests for measles-specific antibodies, CT, MRI, and analysis of the CSF. The management of measles-induced encephalitis mainly revolves around prevention against contracting the disease and providing supportive care if acquired. The administration of the measles vaccine is the major means of preventing this disease in childhood. Two doses are required to achieve sufficient immunity against measles, the first at the age of 12–15 months and the second at 4–6 years of age. Supportive care includes administering acetaminophen for fever, oral rehydrating salt (ORS) for diarrhea and vomiting, antibiotics for otitis media and pneumonia, and using anti-epileptics such as sodium valproate for seizures. Vitamin A can be given to prevent severe effects in children. The specific treatment would depend on the type of encephalitis the patient has developed.

## 1. Introduction

The measles virus, a single-stranded RNA virus, belongs to the paramyxovirus family and is well-known throughout the world for causing measles. In some individuals, it may eventually lead to the development of a variety of complications, some of which could be fatal. In recent years, measles infection has become of prime concern due to the fact that despite the wide availability of effective vaccines against the virus, a sudden upsurge in cases of measles was noted by the World Health Organization (WHO) towards the end of 2019 in states of America with a previously recognized eradication of the virus [1,2]. From the experts’ points of view, the negligence regarding administration of the measles vaccine observed during the recent coronavirus disease (COVID-19) outbreak might be one of the causative factors along with the declining effectiveness of the measles vaccine [1]. The US Centers for Disease Control and Prevention (CDC) reported 16 new cases of measles in September 2021, most of which were found in Afghan refugees and US citizens [3]. In October 2021, Nigeria, Pakistan, Somalia, and India topped the list of countries reporting the highest number of measles cases [2]. Since the emergence of COVID-19, there have been drastic shifts in interest of researchers, scientists, news reporters, and various non-government organizations (NGOs) from already prevalent infectious diseases to the sudden eruption of this novel and peculiar COVID-19 virus and its associated lethality. The virus attracted all the attention and thereby lifted necessary focus from other infective agents, leading to the postponement of previously scheduled vaccination campaigns for measles [2,4]. To date, measles remains the most contagious disease around the world, with an R0 (basic reproduction number) ranking of 12–18, meaning an infected person is likely to spread the disease to an average of 12–18 people (in a totally susceptible population). The high contraction rate of measles poses a major threat to scientists to come up with efficient methods for wisely tackling the outbreak [5].

## 2. Pathogenesis

The measles virus, acquired via aerosol droplets, has an increased propensity for invading the dendritic and alveolar macrophages of the host’s respiratory tract [6]. The process is initiated and fueled by the presence of signaling lymphocytic activation molecule (CDw150) and Nectin-4 on human immune cells and epithelial cells respectively, allowing efficient interaction with the virus [6]. This interaction between the receptors and the virus is crucial for permitting entry of the virus into the cell. Once the invasion is complete, the virus may further proceed to enter other organs through the bloodstream and start replicating in the lymphocytes. Evidence from multiple research studies signify that the virus is highly capable of causing major disruptions to the host’s immune mechanisms, which can render the person increasingly susceptible to opportunistic infections along with the decreased capacity to fight them. This immunomodulation includes decreased functional capacity and count of CD4+ and CD8+ T lymphocytes and reduced production of Interleukin-12 (IL-12), which is responsible for initiating T-helper 1 responses [7].

## 3. Signs and Symptoms

The incubation period, which refers to the time from exposure to the onset of the first symptom, is approximately 11 to 12 days in measles with fever, cough, coryza, Koplik spots and measles rash being the most common presentations [8]. Koplik spots are blue-white spots that usually appear before the morbilliform rash on the buccal mucosa. The measles rash is characterized by a maculopapular eruption beginning from the neck, involving the face and later adopting a centrifugal spread pattern to involve upper and lower extremities as well as the trunk [8]. The infectivity period of measles, which is the duration with the highest chance of the transmission of the infective agent, is 4 days before the onset of rash and 4 days after the rash [8]. Though the primary measles infection usually presents with pyrexia, coryza and maculopapular rash, the alarm bells ring when an individual infected with measles starts developing neurological symptoms secondary to viral invasion of the neurons. Neurological and respiratory complications have proved to be important and the most common causes of death from measles [9]. These complications were more commonly observed in children younger than 5 years and adults, as well as a few immunocompromised or debilitated individuals [8]. Diarrhea and otitis media are other two well-recognized complications of measles [10].

## 4. Encephalitis and Its Types

Encephalitis, which is the inflammation of the brain parenchyma, is a rare but devastating complication of measles, denoting that the virus has breached the neuronal membrane or, undergoing a possible epitope matching phenomenon, the virus might have triggered an inflammatory response in the host. Measles-induced encephalitis has four distinct presentations requiring different approaches. Encephalitis may either follow an acute course (primary measles encephalitis and acute encephalitis) or have a chronic presentation. Chronic presentation is further subdivided into measles inclusion body encephalitis and subacute sclerosing panencephalitis (SSPE) [11].

Primary measles encephalitis occurs during the ongoing primary measles infection and is likely to present in the first week along with the appearance of the characteristic measles rash [12]. The probability of infected people eventually developing primary measles encephalitis is 1 in 1000, and the associated mortality rate is 10–15% [13]. It is highly likely to be caused by the virus directly invading the neuronal cells [12].

Acute encephalitis due to measles, also known as ‘post-infectious encephalitis’, is coupled with a 20% mortality rate and may manifest with fever, headaches, altered sensorium, and even later development of debilitating neurological deficit [11]. Some studies suggest that the activation of the autoimmune mechanism may be the causative factor, whereas other studies favor direct viral invasion to be the prime cause of encephalitis [14]. Due to the scarcity and reliability of available data, it is hard to state the exact pathogenesis of acute encephalitis.

The third type of measles inclusion body encephalitis (MIBE) also known as subacute measles encephalitis, is more likely to be observed in people with compromised immunity, and the presentation may progress from an initial altered level of consciousness to worsening seizures, eventually leading to established epilepsy and focal neurological deficits such as aphasia (inability to talk), hemiplegia (paralysis of one side of the body), and ataxia (lack of coordination of movements) [12,15]. The mortality rate ascribed to MIBE is 75% [12].

Subacute sclerosing panencephalitis, a chronic demyelinating disease, is a result of persistent viral invasion of the neurons, reactivation of previously acquired measles virus, or an underdeveloped immune system as seen in children younger than 2 years of age [12,16]. Recent molecular studies suggest viral F-protein to be responsible for neurovirulence by undergoing conformational change from a perfusion state to a steadier post-fusion state, thereby promoting healthy viral and host cell interactions [17]. Histopathology of the disease suggests the involvement of oligodendrocytes, astrocytes and endothelial cells showing the presence of inclusion bodies and/or fibrillary tangles [16]. The infective course begins with typical cellular swelling along with nuclear degeneration induced by oxidative stress, and later progresses to demyelination of the neurons [18]. Leukocytes infiltrating the brain parenchyma indicate the presence of an ongoing acute inflammatory response which later exhibits symptoms with respect to the extent of damage that has been done. The symptoms begin with certain behavioral changes and decreased efficiency, and progress toward subsequent cognitive decline and significant motor deficits which can later prove to be fatal. Clinical changes observed in the diagnosis of SSPE are divided into four stages. Changes seen in stage one include behavioral and cognitive decline. Myoclonic jerks, seizures, and dementia are likely to be seen in stage two. Stage three presents with rigidity, extrapyramidal symptoms, and progressive unresponsiveness. Stage four is the last stage and involves coma, persistent vegetative stage, akinetic mutism, and autonomic instability [19]. The mortality rate is around 95% [18].

## 5. Management

### 5.1. Diagnosis

Prompt diagnosis of post-infectious encephalitis is of immense importance, as it has a high tendency of giving rise to devastating sequelae. The disease can be suspected if certain clinical features are present, such as fever, headache, altered level of consciousness, focal neurological deficits, hemiplegia, aphasia, ataxia, paraplegia, or specific signs of inflammatory demyelination such as optic neuritis [20], which signals towards an underlying brain parenchymal injury.

These clinical presentations still prove to be quite ambiguous and are insufficient to make a definitive diagnosis. Hence, a precise history can help move towards the diagnosis. Relevant questions should include the location or residence of the patient and seasonal pattern of occurrence of disease in order to rule out diseases such as Japanese encephalitis, which peaks in the rainy season. A detailed contact history should be taken, which may include any exposure of the patient to an infected individual or any travel or migration from a high-risk measles area. Enquiry related to any exposure to farm animals is also equally crucial to rule out other causes of viral encephalitis, such as flavivirus. Immunization status is sought to exclude encephalitis due to listeriosis, cryptococcus, and cytomegalovirus, as these infections are highly suspected in immunocompromised patients. Occupational history contributes to eliminating work-related causes; for example, Lyme disease or Kyasanur Forest disease [21].

Next comes the physical examination. If conducted thoroughly, it can help to guide the evaluation of the disease in the right direction. Examination of the patient can further suggest that the infection has spread to the brain. This includes general examination of the skin, mucous membranes, lymph nodes, and central nervous system [21]. Some specific findings include positive Kernig’s sign, nuchal rigidity, involuntary movement of muscles [22], low Glasgow Coma Scale (GCS) score, visual defects on ophthalmology, dysarthria, general hypotonia, depressed reflexes [23], photophobia, and speech disturbances [24].

The next step in the workup is to carry out laboratory testing to confirm the diagnosis, and this includes:

- Blood tests [biochemical and hematological] to identify measles-specific antibodies, specifically immunoglobulins IgG and IgM in the serum [12]. Previous research observed greater levels of IgE in serum of children with encephalitis as compared to those with uncomplicated measles [25];
- Nasopharyngeal swabs or throat swabs and urine samples, which may also reveal presence of measles virus via polymerase chain reaction (PCR) [26];
- Chest radiography to look for the spread of measles to the lungs in the form of pneumonia [27];
- Electroencephalography (EEG);
- Computed tomography (CT);
- Magnetic resonance imaging (MRI) of the brain (with contrast);
- Single photon emission computed tomography [SPECT, optional, depending on availability];
- Cerebrospinal fluid (CSF) analysis by lumbar puncture and cells, biochemistry, and molecular diagnostic tests (PCR)—Analysis of CSF fluid would show lymphocytic pleocytosis with normal glucose, normal or mildly raised protein, and measles virus RNA would be seen on PCR;
- Brain biopsy (in some cases) [21].

### 5.2. SSPE

This is a rare and fatal complication of measles infection, and a timely diagnosis is crucial. Diagnosis of SSPE is made using Dyken’s criteria which consists of two major and four minor criteria. According to the major criteria, firstly, antibodies against measles in cerebrospinal fluid are increased in levels greater than or equal to the ratio of 1:4, or antibodies found in serum are greater than or equal to 1:256; and secondly, the patient presents with typical or atypical symptoms of measles. Symptoms are called typical if the disease is rapidly growing or if the disease is long lasting and occurring on and off. Atypical symptoms consist of fits, extended stage one of disease, or an odd age of presentation that occurs in a newborn or an adult. The following are the minor criteria: (1) EEG showing periodic, generalized, bilaterally synchronous and symmetrical high-amplitude slow waves which occur regularly after every 5–15 s, which are known as periodic slow-wave complexes or “Radermecker” complexes; (2) immunoglobulin IgG levels in CSF which are higher than 20% of the sum of all proteins in CSF; (3) detection of inflammatory changes in the meninges and cerebral parenchyma necrotizing leukoencephalitis with diffuse demyelination, viral inclusion bodies in neurons, oligodendrocytes and astrocytes, neuronal loss, and astrocytosis on brain biopsy that are distinctive for SSPE; and (4) specialized molecular diagnostic test to identify the wild-type measles virus mutated genome.

SSPE is diagnosed if the patient has two major and at least one minor criteria [19].

## 6. Treatment

The treatment of measles and its complications mainly revolves around preventative measures and providing supportive care if acquired. Firstly, infected individuals must be isolated, and all protective measures such as the use of personal protective equipment (PPE) such as N-95 masks, gloves and sterilized gowns by health care professionals should be implemented as per protocol. Proper handwashing technique is also encouraged.

## 7. Prevention and Control

The prevention of the measles virus through vaccination is the cornerstone of its treatment. According to ACIP (Advisory Committee on Immunization Practices), it is recommended to obtain two doses of a live attenuated MMR (measles, mumps and rubella) vaccine, with the first dose administered from 12 to 15 months of age and the second one from an age of 4 to 6 years [28]. In areas where incidence and death rate from measles is high, vaccination can be started earlier, i.e., at the age of 9 months [29].

Research shows that the vaccine is 95% effective after its first dose and 96% effective after the second [30]. Before the development of vaccine for measles, the number of cases remained almost unchanged until 1963, and declined for the first time in 1965. The death rate fell from 0.23 per 100,000 in 1963 to 0.065 per 100,000 population between 1965 to 1970 after the introduction of vaccine [31].

The measles vaccine works by stimulating the proliferation of both antibodies and CD T4 cells, providing immunity. However, it is less effective and short-lived compared to immunity induced by the wild virus itself [32], as the antibodies produced as a result of vaccination may not reach their optimal levels. Therefore, giving immunoglobulins passively can help preserve the antibody pool [11].

One study also stated that if immunoglobulins are administered within 72 h of exposure to measles, there is a 75% possibility of preventing the infection. The dose to be given in the first 6 days of exposure is 0.08–0.1 cc/lb [33].

Specific medications for measles do not exist. Only symptomatic treatment is given for the alleviation of symptoms such as fever, cough, coryza, conjunctivitis, and mouth ulcers [29]. Patients are advised to rest, keep themselves hydrated, and take acetaminophen to bring down their temperatures if febrile [34]. Measles-related loose stool, vomiting, and decreased appetite can lead to malnourishment, which can be cured by providing dietary support. In case dehydration occurs, oral rehydration salts are given, and breastfeeding mothers are advised to continue feeding to maintain hydration [29]. Antibiotics are recommended as treatment if the disease progresses and causes pneumonia or otitis media [35]. Commonly, divalproex sodium is used in case where seizures develop [36].

Administration of vitamin A is recommended in all acute cases, either as capsules or in liquid form. It is known to decrease the risk of measles-related death by 87% in children less than 2 years of age. In addition to this, it is effective in lowering the timespan of diarrhea by 2 days and of fever by 1 day in children of all age groups [37]. In infants less than 6 months of age 50,000 IU is given when diagnosed and 50,000 IU administered again the next day. If the infant is 6 to 12 months of age, a dose of 100,000 IU is given on diagnosis and another dose of 100,000 IU is given the next day. In children aged 12 months and above, the dose increases to 200,000 IU at diagnosis and another 200,000 IU is given the next day [38]. A review focusing on vitamin A and its impact on measles-related morbidity and mortality suggests a marked reduction (64%) in children under the age of two, with a powerful impact on measles-associated complications [39]. This effect was noted after vitamin A administration for two continuous days.

Patients afflicted with the measles virus can progress toward developing serious complications including diarrhea, otitis media, pneumonia, and encephalitis, which must be addressed promptly. In the United States, one or more complications occurred in around 30% of cases of measles from 1987 to 2000 [8]. Approximately one to three children out of 1000 affected by measles die from respiratory and neurologic complications [10], and so they must be cared for. Of those infected with measles, one per every 1000 children suffers from encephalitis, which can result in convulsions, hearing impairment, and mental retardation [10].

A study conducted in France in 2013 showed that 4 out of 32 measles infected patients developed measles-induced encephalitis, with three cases of post-infectious encephalitis and one case of measles inclusion body encephalitis [40]. Hence, this complication should be dealt with immediately. From the facts and figures stated above, infected people are prone to develop ominous complications if immediate and effective treatment measures are not adopted. Hence, emphasis is placed on efficiently dealing with the disease in its initial stages.

Since there are four types of measles-induced encephalitis and the types involve comparatively different pathologies and disease severities, the treatment plans for each type also differ. Treatment of measles-induced encephalitis depends on its type. The types and respective treatment for each are as follows:

1. Primary measles encephalitis. Treatment majorly involves the curing of symptoms [12]. Patients should be isolated to prevent transmission to other immunocompromised patients, and in cases involving a pediatric age group, patients should be shifted to a pediatric intensive care unit (PICU) if needed [13].
2. Acute post-measles encephalitis. As this form of encephalitis is immune-mediated, corticosteroids are given as first-line treatment [12], which at low levels suppress inflammation by inhibiting nuclear factor-κB (NF-κB), activator protein-1 (AP-1), and MAP kinase phosphatase-1 (MKP-1), which are all involved in inflammatory response and, at higher levels, increase transcription of anti-inflammatory genes. If steroids fail to work, IgG can be given intravenously [41]. Maternal antibodies can protect a child until 15 months at most, after which they fade away, so injecting immunoglobulins can prove to be very beneficial [42].
3. Measles inclusion body encephalitis. Supportive treatment [12] is given; however, a case report showed that intravenously administered ribavirin promoted improvement in a 4-year-old with ALL (Acute Lymphocytic Leukemia) [24].
4. Subacute sclerosing panencephalitis. There is still no proven treatment for SSPE, but a combination of ribavirin (RBV), Inosiplex (Isoprinosine), and intraventricular interferon-a [IFN-a] has demonstrated to be the most effective way to deal with the disease and shorten the disease course [43]. Ribavirin is an antiviral drug, and it acts as a nucleic acid analogue, blocking the enzyme RNA-dependent RNA polymerase and thus hampering viral RNA and protein synthesis [44]. The results of study found that the injection of high dose intravenous ribavirin into CSF showed betterment of CNS manifestations [45]. Inosiplex and Interferon-a function as antivirals and immunosuppressants [46,47]. A case study of a 10-year-old Japanese boy demonstrated that the advancement of SSPE can be avoided by using intraventricular interferon-α. High levels of T helper 17 (TH-17) cells were shown on a flow-cytometric analysis with the use of intraventricular interferon-α [48]. According to a case report, the combination of ribavirin with Interferon-alpha and Inosiplex proved to be a good treatment option with positive responses [49]. Another clinical trial showed that cimetidine, a H2 histamine blocker agent, might assist in controlling the development of the disease by suppressing histamine mediated T lymphocytes activation [50]. A case report of a 13-year-old boy who developed myoclonus as a result of SSPE showed improvement with the usage of carbamazepine, an antiepileptic, before going into a vegetative state [51].

Other treatments are also available but not so widely practiced, as the data on their efficacy are yet not sufficient. Transcription or replication inhibitors of the virus RNA can transiently prevent the growth of the virus. Small interfering RNAs (siRNAs) or shRNAs are synthetic oligonucleotides which can halt the production of polymerase complex, which controls the transcription of viral RNA. The process of transcription also briefly requires heat shock proteins, such as the heat shock protein 90 (HSP90). Geldanamycin and derivatives such as 17-DMAG (17-Dimethylaminoethylamino-17-Demethoxygeldanamycin) block the activity of these proteins and might impede the transcription process [11]. Nucleoside analogs including Remdesivir (GS-5734) and R1479 may also block polymerase activity of the measles virus. Moreover, the compound 16677 (1-methyl-3-trifluoromethyl-5-pyrazolecarboxylic acid) acts as non-nucleoside inhibitor of the RNA-dependent RNA polymerase complex and can also help stop the transcription and replication of the measles virus [11].

Since F protein of host cells and H protein of SSPE are involved in the fusion of the measles virus to host cells and facilitate their entry into host cells, antibodies against H protein can inhibit this fusion. There is evidence that Aprepitant, which is an antagonist of the neurokinin-1 receptor (which is also known to aid in this fusion), has lowered the spread of the virus in measles-containing vaccines to the brain [11]. As the name suggests, the fusion inhibitor peptide (FIP) 2-D-Phe-L-Phe-Gly also prevents the fusion and entry of the virus into host cells. In addition, HRC-derived peptides work by halting the F protein in its post-triggering stage, hence stopping the fusion process [11].

## 8. Disease Controlling Initiatives

CDC and WHO have successfully reduced the global burden of infectious diseases such as measles in countries such as the United States for the last 20 years by strategizing plans to lower its transmissibility [52,53].

The Measles Outbreaks Strategic Response Plan 2021–2023 was formulated by the WHO for the prevention of measles and to promote the readiness to fight measles outbreaks. It aims to conduct risk assessments every year to help formulate effective strategies against measles, to look into measles outbreaks, and to take immediate action to curb the spread of disease. It also plans to look over the main ground of the disease to fill in the immunity gap and avoid future episodes by taking appropriate measures. Surveillance of the outbreaks in 2022 can assist in identifying what is lacking in the immunization system, and suitable approaches can be thought of to bridge the immunity gaps [2].

CDC started the “Measles and Rubella Initiative” which is intended to carry out, observe, and assess measles and rubella vaccination programs with the goal of eliminating both diseases. The plan proposes recommendations based on its operational research studies to direct activities aimed at preventing these diseases. It also aims to supply technical aids for inspecting measles and rubella breakouts, for monitoring these outbreaks, and to check vaccination status. In addition to this, it assists in initiating measles and rubella monitoring programs in different areas where these diseases have a high incidence. Furthermore, it plays the role of a reference laboratory all over the world and supplies assets on a national level. It contributes in the gathering and shipping of blood samples to test for measles using real-time polymerase chain reaction (RT-PCR) [54]. It was due to these efforts that the weekly report of CDC’s Morbidity and Mortality in November 2021 showed that recently, measles deaths have fallen by 94% since the beginning of 2020.

## 9. Conclusions

Immediate recognition and diagnosis of the disease can only be possible if healthcare workers are preemptively educated with detailed information regarding the various presentations of complaints associated with measles and its complications, such as measles-induced encephalitis. Hence, being aware of the different types of measles presentations and thereby discouraging delayed diagnosis as well as initiating appropriate treatment may help in the prevention of the development of deleterious complications. Nevertheless, herd immunity remains the prime goal. Considering the highly infectious nature of the virus, attempts at executing effective and well-planned herd immunity programs is of immense importance. This can only be achieved if adequate consideration is given to prevailing diseases other than COVID-19. The realization of the fact that the measles virus is highly capable of triggering vicious responses in the brain will reduce the tendency to overlook measles vaccination campaigns, especially during the COVID-19 outbreaks. If the recent innovations and plans suggested to control measles and its related complications are carried out under strict supervision, there will be high chances of successfully eradicating the virus.

## Data Availability

Not applicable.
